# Modeling and Molecular Dynamics of HPA-1a and -1b Polymorphisms: Effects on the Structure of the β3 Subunit of the αIIbβ3 Integrin

**DOI:** 10.1371/journal.pone.0047304

**Published:** 2012-11-14

**Authors:** Vincent Jallu, Pierre Poulain, Patrick F. J. Fuchs, Cecile Kaplan, Alexandre G. de Brevern

**Affiliations:** 1 Laboratoire d’Immunologie Plaquettaire, INTS, Paris, France; 2 INSERM, U665, DSIMB, Paris, France; 3 Univ Paris Diderot, Sorbonne Paris Cité, UMR-S665, Paris, France; 4 Institut National de la Transfusion Sanguine, Paris, France; 5 Laboratoire d'Excellence GR-Ex, Paris, France; National Cerebral and Cardiovascular Center, Japan

## Abstract

**Background:**

The HPA-1 alloimmune system carried by the platelet integrin αIIbβ3 is the primary cause of alloimmune thrombocytopenia in Caucasians and the HPA-1b allele might be a risk factor for thrombosis. HPA-1a and -1b alleles are defined by a leucine and a proline, respectively, at position 33 in the β3 subunit. Although the structure of αIIbβ3 is available, little is known about structural effects of the L33P substitution and its consequences on immune response and integrin functions.

**Methodology/Principal Findings:**

A complete 3D model of the L33-β3 extracellular domain was built and a P33 model was obtained by *in silico* mutagenesis. We then performed molecular dynamics simulations. Analyses focused on the PSI, I-EGF-1, and I-EGF-2 domains and confirmed higher exposure of residue 33 in the L33 β3 form. These analyses also showed major structural flexibility of all three domains in both forms, but increased flexibility in the P33 β3 form. The L33P substitution does not alter the local structure (residues 33 to 35) of the PSI domain, but modifies the structural equilibrium of the three domains.

**Conclusions:**

These results provide a better understanding of HPA-1 epitopes complexity and alloimmunization prevalence of HPA-1a. P33 gain of structure flexibility in the β3 knee may explain the increased adhesion capacity of HPA-1b platelets and the associated thrombotic risk. Our study provides important new insights into the relationship between HPA-1 variants and β3 structure that suggest possible effects on the alloimmune response and platelet function.

## Introduction

The human platelet antigen (HPA)-1 alloimmune system, carried by the platelet integrin αIIbβ3 (GP IIbIIIa), is of major clinical interest. It is the first cause of alloimmune thrombocytopenia in Caucasian populations [1], [2] and the allele HPA-1b may be a risk factor for thrombosis [3]. This alloantigenic system is characterized by a leucine-to-proline substitution in position 33 of the mature β3 subunit of the αIIbβ3 integrin, changing allele HPA-1a into HPA-1b [4]. Nonetheless, alloimmune responses to the HPA-1a and HPA-1b variants have been shown to be complex [5], [6] and to differ in their frequencies [7]. Similarly, association between the HPA-1b variant and a thrombosis risk factor is still debated [8], [9], and, although experimental data have suggested enhanced platelet functions [10], the mechanism that potentially leads to thrombosis remains to be elucidated. One potential mechanism may operate through an alteration of the molecular structure of β3. To compare the structural effects of the HPA-1a and HPA-1b variants, we built 3D models of the L33 and P33 β3 forms from an αIIbβ3 structure available in the Protein Data Bank (PDB) [11], [12]. Molecular dynamics (MD) simulations for a cumulated time of 300 ns were performed for both β3 forms. Protein blocks (PB) analyses [13] were combined with classical MD trajectory analyses to understand how local polymorphisms can affect the dynamical behavior of β3. To our knowledge, this is the first study demonstrating that the PSI, I-EGF-1 and I-EGF-2 domains of β3 are highly flexible and that the L33P substitution modifies the local structural equilibrium.

Based on *in silico* modeling and MD simulations, we compared the dynamical structures of the HPA-1b form (Pro33) of β3 with its HPA-1a (Leu33) form. Surface exposure, and contacts of the polymorphic residues as well as interactions between the PSI, I-EGF-1, and I-EGF-2 domains are modified. These results therefore provide important new structural clues that can explain HPA-1a and -1b allelic differences in immune response and thrombogenesis. This powerful methodology is easily transposable to most mutations resulting in hereditary or alloimmune pathologies.

## Materials and Methods

### Modeling of β3 Structures

The initial β3 structure was taken from the 2.55 Å resolution crystal structure of integrin αIIbβ3 [12] from the PBD (PDB ID 3FCS). A complete structural model was obtained with MODELLER v9.6 software [14] by filling the 75–78 and 477–482 crystallographic gaps and by reverting the P688C mutation. For this modeling step, structural templates were taken from the αIIbβ3 (PDB ID 3FCS) and the αvβ3 complexes (PDB ID 3IJE, 2.90 Å resolution) [15]. We also took care to maintain the 28 disulfide bonds. The L33P substitution was done using PyMOL software [16]. The final models were composed of 690 residues that correspond to the β3 ectodomain. In what follows, the PSI domain of β3 is defined from residue 1 to 56, the I-EGF-1 domain from residue 434 to 472 and the I-EGF-2 domain from residue 473 to 522. According to the supplementary data in reference [12], residues 434 and 435 should be assigned to the PSI domain, but for the sake of clarity were assigned to the I-EGF-1 domain here.

### Molecular Dynamics Simulations

MD simulations were performed using GROMACS 4.0.7 software [17] with the OPLS-AA force-field [18]. L33 and P33 forms of β3 were soaked in a rhombic dodecahedral simulation box with 60,622 TIP3P water molecules and 28 Na^+^ ions. The distance between any atom of the protein and the box edges was set to at least 10 Å. The total energy of the system was minimized twice (before and after the addition of the ions) with a steepest descent algorithm. MD simulations were run under the NPT thermodynamic ensemble and periodic boundary conditions were applied in all directions. We used the weak coupling algorithm of Berendsen [19] to maintain the system at a constant physiological temperature of 310 K using a coupling constant of 0.1 ps (protein and water ions separately). Pressure was held constant using the Berendsen algorithm [19] at 1 atm with a coupling constant of 1 ps. Water molecules were kept rigid using the SETTLE algorithm [20]. All other bond lengths were constrained with the LINCS algorithm [21], allowing a 2 fs time step. We used a short-range coulombic and van der Waals cut-off of 10 Å and calculated the long-range electrostatic interactions using the smooth particle mesh Ewald (PME) algorithm [22], [23] with a grid spacing of 1.2 Å and an interpolation order of 4. The neighbor list was updated every 10 steps. After a 1 ns equilibration (with position restraints on the protein), each system was simulated for 50 ns. For both systems, five 50 ns simulations were performed (using different initial velocities) and one was extended until 100 ns for a total simulation time of 300 ns. Molecular conformations were saved every 100 ps for further analysis.

### Trajectory Analyses

The first 5 ns of each MD simulation were discarded and trajectory analyses were conducted on a set of 2,749 MD snapshots for each system and were performed with the GROMACS software and in-house Python and R scripts. Root mean square deviations (RMSD) and root mean square fluctuations (RMSF) were calculated on Cα atoms only. To analyze the number of contacts, two residues were defined as contacting each other when the distance between their Cα atoms was less or equal to 8 Å [24]. The absolute accessible surface area (ASA) was computed using GROMACS software. The relative accessible surface area (rASA) was obtained by normalizing the absolute ASA to the ASA calculated on an extended peptide Ala-X-Ala (where X stands for the amino acid in question), in this case 183 Å^2^ and 142 Å^2^ for Leu and Pro, respectively [25]. Distances between residue 33 and the I-EGF-1 and I-EGF-2 domains were computed as the distance between the center of mass of the atoms in residue 33, and the I-EGF-1 and I-EGF-2 domains.

Principal component analysis (PCA, also called essential dynamics) is a convenient method for filtering a trajectory [26]. The principle is to align each snapshot to a reference structure and to calculate the covariance matrix. This matrix gives information on how each pair of atoms fluctuates, in a correlated or in an uncorrelated way. By diagonalizing this matrix, a set of collective motions (*i.e.* atoms that move together during a trajectory) is obtained in the form of eigenvectors, and the eigenvalues give the amplitude of each motion. In general, only the first 10 eigenvectors (also called modes) are relevant, all subsequent ones are associated with noise (*i.e.* vibrations). The first two eigenvectors then represent the two collective motions with the highest amplitude. Projecting a trajectory on these two modes is a convenient way to quantify the motion that a protein undergoes. We performed PCA using GROMACS software.

### Protein Blocks Analysis

PBs [27] are a structural alphabet composed of 16 local prototypes. Each specific PB is characterized by the φ, ψ dihedral angles of five consecutive residues. Obtained through an unsupervised training approach performed on a representative non-redundant databank, PBs give a reasonable approximation of all local protein 3D structures [13]. PBs are very efficient in modeling tasks such as protein superimpositions [28] and MD analyses [29]. They are labeled from *a* to *p* (see Figure 1 in ref. [30]): the PBs *m* and *d* can be roughly described as prototypes for α-helix and central β-strand, respectively. PBs *a* to *c* primarily represent β-strand N-caps and PBs *e* and *f*, β-strand C-caps; PBs *a* to *j* are specific to coils, PBs *k* and *l* to α-helix N-caps, and PBs *n* to *p* to α-helix C-caps. PB [31] assignment was carried out using an in-house program written in C (available at http://www.dsimb.inserm.fr/~debrevern/DOWN/LECT/).

**Figure 1:**
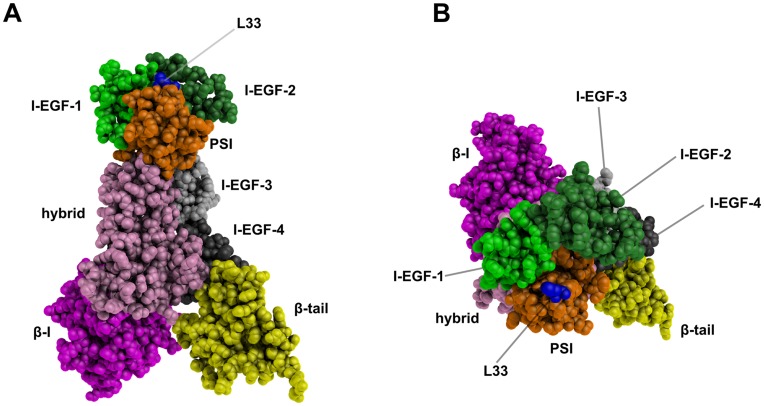
Ectodomain 3D structure model of the L33 β3 form. A side view of β3 integrin (A) and an apical view of the β3 knee (B) are shown. Domains are differently colored and labeled and the L33 residue is shown in blue. These static views illustrate the HPA-1 polymorphic site that is located at the top of the β3 knee. These representations were generated using PyMOL software [16].

PB assignments are done for every residue of the β3 protein and for every snapshot extracted from the MD simulations. The equivalent number of PBs [13] (*N_eq_*) is a statistical measurement similar to entropy and represents the average number of PBs a given residue takes. *N_eq_* is calculated as follows:
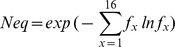
Where 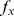 is the probability of PB *x*. A *N_eq_* value of 1 indicates that only one type of PB is observed, while a value of 16 is equivalent to a random distribution. To underline the main differences between the two proteins, Δ*N_eq_* values corresponding to the absolute difference between *N_eq_* from L33 and P33 simulations were computed. Differences were considered significant when at least one of the *N_eq_* was less than 2.5 and Δ*N_eq_* was greater than 1.5.

## Results

### Structure Modeling of the L33 and P33 β3 Variants and Electrostatic Comparisons

The L33P substitution is responsible for the HPA-1 system, clinically the most important HPA system described to date. Some experimental data suggest that the L33P substitution affects platelet functions [10]. Our goal was to assess the structural origins of these clinical observations by analyzing the effect of the L33P substitution on the structure of the β3 subunit. To do so, we built 3D models of the β3 allelic forms L33 and P33 (see Materials and Methods).

A RMSD of 1.8 Å revealed no significant structure divergence between our β3 models (690 residues) and the experimental β3 structure associated with αIIb (PDB ID 3FCS). The 3D model of the L33 β3 form is shown in Figure 1. The polymorphic site (residue 33), localized in the PSI domain, is in close vicinity to the I-EGF-1 and I-EGF-2 domains. Because β3, and the PSI domain in particular, are rich in disulfide bonds [32], all disulfide bonds in the models were carefully checked.

Electrostatic maps projected on van der Vaals molecular surfaces are valuable tools for studying the impact of mutations [33]. Figure S1 in supplementary material shows electrostatic maps projected on the 3D models of the L33 and P33 allelic forms viewed from the β3 knee. They show a similar overall negative charge of the β3 knee area for both alleles. The L33P substitution did not induce any significant electrostatic changes or structure modifications. L33 and P33 structures were therefore further studied by MD simulations.

### Molecular Dynamics Simulations Reveal High β3 Structure Flexibility

Four independent MD simulations of 50 ns were performed for each β3 form. A fifth MD of 100 ns was carried out to ensure stability of each model over a longer simulation time. After 5 ns, all simulations converged to a steady state that was maintained up to 50 or 100 ns, indicating that models were stable during simulations (Figure S2A). For both β3 variants, statistical analyses were performed on 2,749 structures. The PSI domain is in close vicinity to the I-EGF-1 and I-EGF-2 domains that are at the epicenter of conformational changes that occur upon integrin activation [12]. Therefore, statistical analyses presented here were restricted to the PSI (residues 1–56), I-EGF-1 (residues 434–472), and I-EGF-2 (residues 473–522) domains (see Figure 1).

**Figure 2:**
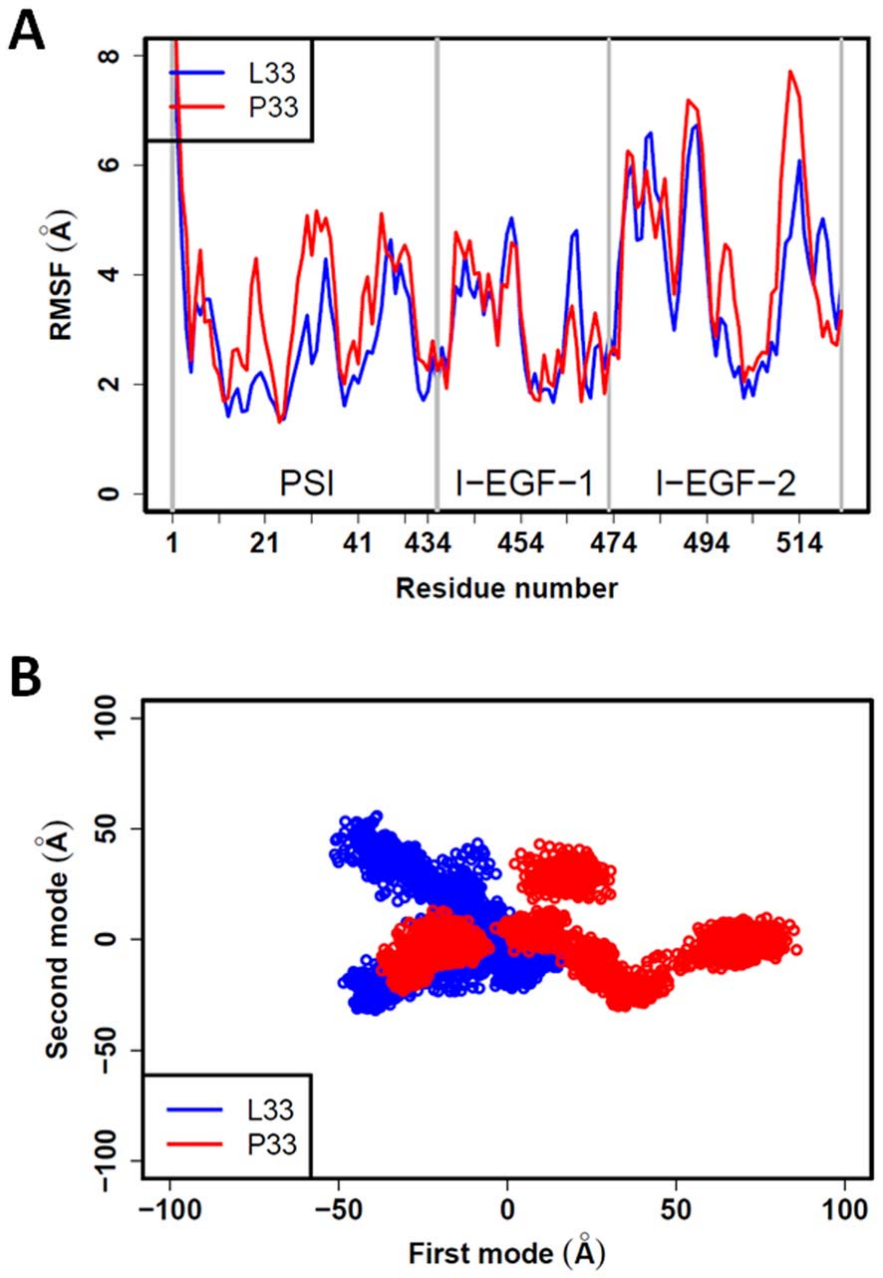
Structural flexibility of domains PSI, I-EGF-1, and I-EGF-2. (A) RMSF values calculated for each residue of the L33 (blue line) and the P33 (red line) forms of β3. Both structures show high flexibility. (B) Atom movements analyzed by PCA are projected on the first two modes. The P33 structure movements (red cloud of dots) are higher than that measured for the L33 structure (blue cloud of dots) for the first mode. No significant differences were observed for the second mode. The proline increased the structural flexibility of the PSI, I-EGF-1 and I-EGF-2 domains. The first and second modes represent 45% and 13% of the informativeness of the data.

All five simulations of each β3 form led to similar results (Figure S2A), but mean RMSD values of 4.7±0.7 Å for the L33 β3 form, and of 7.2±1.0 Å for the P33 form, suggested structural differences between the L33 and P33 forms (Figure S2B). Structural flexibility was then assessed by computing the RMSF values for each residue in the PSI, I-EGF-1, and I-EGF-2 domains (Figure 2A). RMSF variation showed highly similar behavior in the L33 and P33 structures and values of up to 6 Å revealed significant flexibility. For most residues in the PSI domain, RMSF values in the P33 β3 form were higher than in the L33 β3 form.

Structural flexibility was also assessed using PCA. Figure 2B compares projections of the L33 and P33 structures obtained for all simulations for the two first modes, which correspond to the largest collective motions. For the first mode, the spread of the P33 structures was greater than the one obtained for L33, demonstrating that the structure of the P33 β3 variant is more flexible. In the second mode, the spreads were similar. The PCA analysis therefore indicated that the proline in position 33 introduces flexibility in the PSI, I-EGF-1, and I-EGF-2 domains of the β3 structure.

### The L33P Substitution Modifies the Local Structure Equilibrium

**Figure 3:**
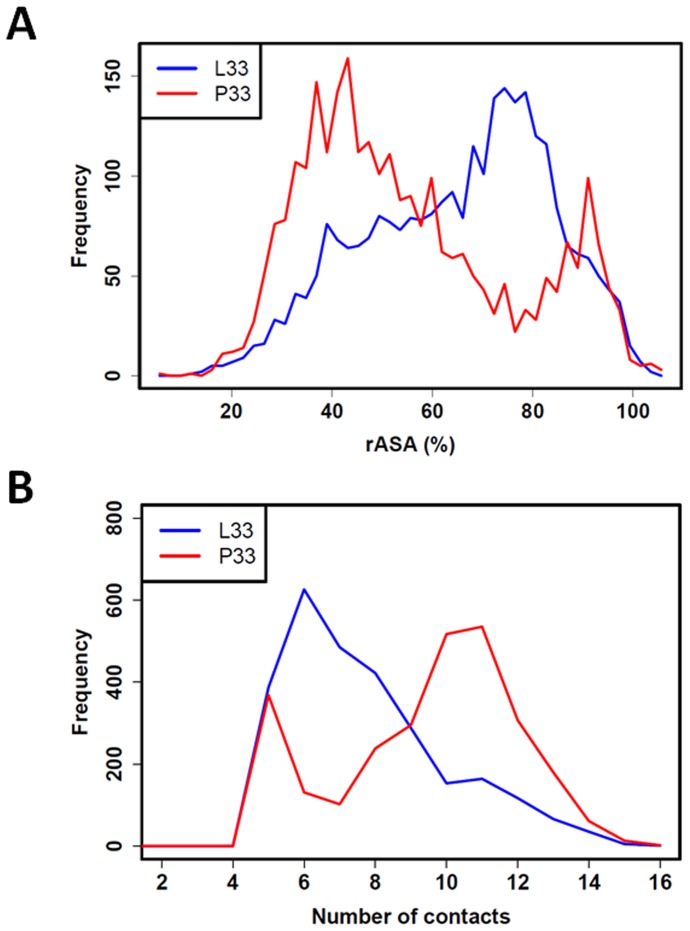
rASA and number of contacts for L33 and P33. Panels A and B respectively show rASA and number of contact distributions for L33 (blue) and P33 (red). These distributions show that L33 is more exposed and establishes fewer contacts than P33.

In regard to the antigenicity of the two forms, surface exposures of L33 and P33 were compared by measuring their relative ASA (rASA). rASA of L33 and P33 ranged between 20 and 100% indicating that they are both exposed to solvent (Figure 3A). Regarding their respective rASA distributions, L33 showed high exposure (peak at 75–80%) more frequently than P33 (peak at 40–45%).

Figure 3B shows distributions of the number of contacts that L33 and P33 established with neighboring residues during MD simulations. L33 and P33 could both form contacts with 4 to 15 residues but on average, L33 had fewer contacts (4 to 10) than P33 (8 to 14). Regarding contact distribution with each domain, the difference mainly resided in a loss of contacts between L33 and residues from the I-EGF-2 domain and, to a lesser extent, residues from the I-EGF-1 domain (Figure S3). The number and distribution of contacts with residues from the PSI domain were similar for both L33 and P33 forms.

**Figure 4:**
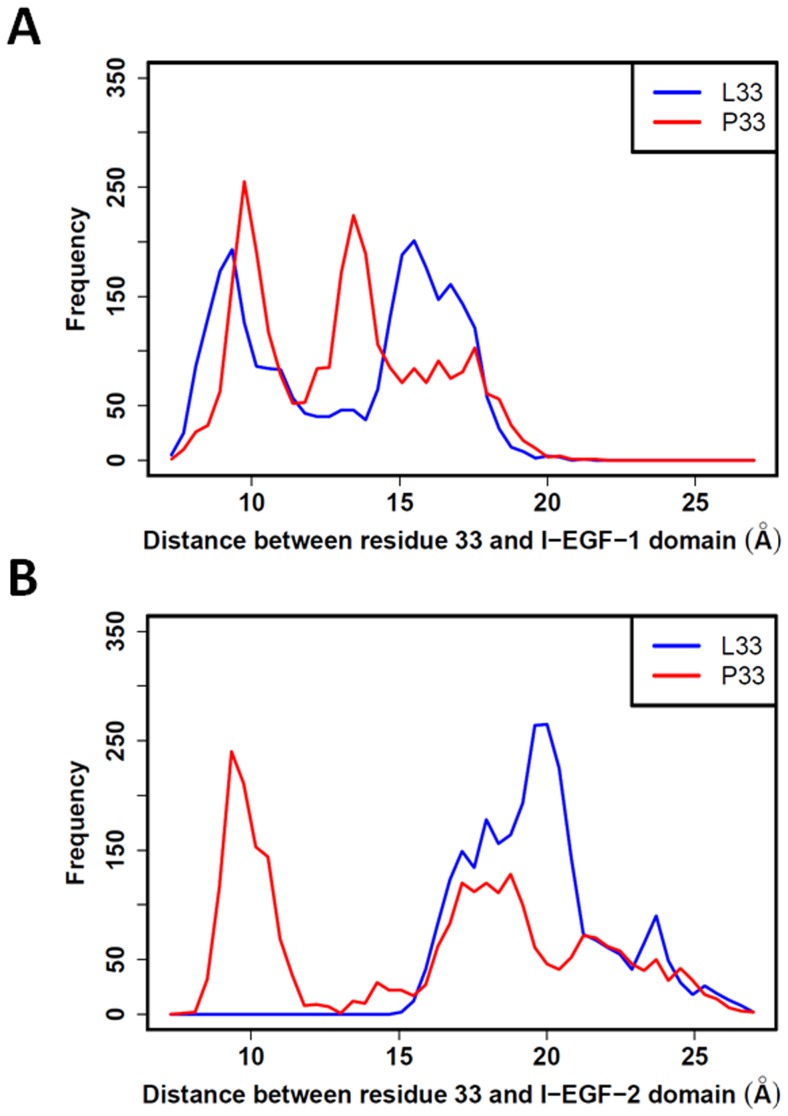
L33 and P33 distances to domains I-EGF-1 and I-EGF-2. Center-of-mass distances were measured between L33 (blue line) or P33 (red line) and the I-EGF-1 (A) or I-EGF-2 (B) domains. No significant differences in distribution were observed regarding the I-EGF-1 domain. However, P33 is close to the I-EGF-2 domain (≈10 Å) while L33 always remains farther than 15 Å.

Finally, we studied the distribution of the distances between the centers of mass of residues L33 and P33, with the centers of mass of I-EGF-1 and I-EGF-2 domains (Figure 4). For the I-EGF-1 domain (Figure 4A), both L33 and P33 showed two peaks of frequency with only slight differences. More interesting are the distribution profiles obtained for the I-EGF-2 domain. P33 clearly adopted a position close to the center of mass of the I-EGF-2 domain with a distance around 10 Å that is never reached by L33 (Figure 4B). In both cases, center-of-mass distances measured for L33 and P33 are coherent with the distributions of their respective numbers of contact with residues from the I-EGF-1 and I-EGF-2 domains (see above).

The PSI, I-EGF-1, and I-EGF-2 domains show a very dynamic structure, and the L33P substitution modifies the local structure equilibrium.

### Analyses of Local Conformation of the PSI, I-EGF-1 and I-EGF-2 Domains using the Protein Blocks Method

**Figure 5:**
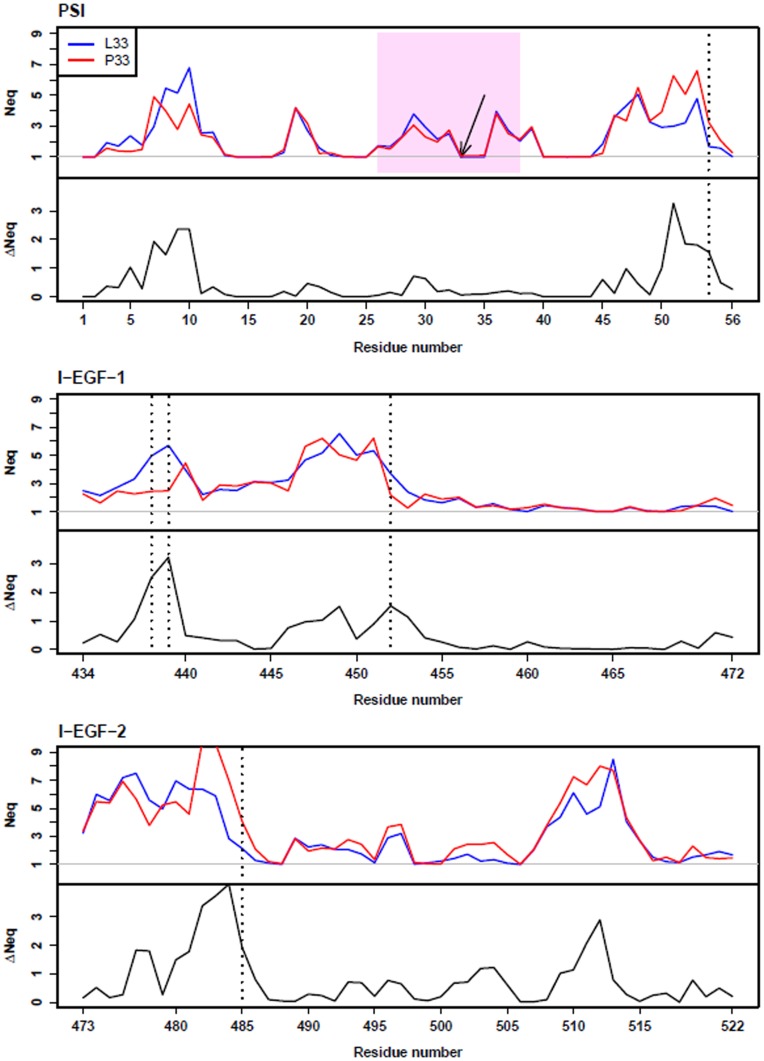
Local structure and *N_eq_* analyses. *N_eq_* values (average number of PBs weighted by their frequencies, see Materials and Methods) of residues from the PSI, I-EGF-1, and I-EGF-2 domains were computed for L33 (blue lines) and P33 (red lines) β3 forms. For each domain, the absolute value of *N_eq_* L33 and *N_eq_* P33 differences (Δ*N_eq_*) is also plotted (black lines). The sequence of the C26–C38 loop in the PSI domain is highlighted (pink-shaded area). The horizontal gray lines indicate an *N_eq_* value of 1. A *N_eq_* value of 1 indicates strong structure rigidity since only one PB is adopted by the residue; above 1, flexibility is proportional to the *N_eq_* value. *N_eq_* analyses demonstrated that the three domain structures are highly flexible, in particular, PSI has four small interspersed rigid areas. L33 and P33 (indicated by an arrow), belong to a rigid stretch (residues 33 to 35). Residues whose Δ*N_eq_* was significantly modified (see Materials and Methods) by the L33P substitution are identified by a vertical dotted line.

The PB representation (see Materials and Methods) was used to analyze local geometrical structures of the PSI, I-EGF-1, and I-EGF-2 domains induced by the L33P substitution. To statistically assess structure alterations, the equivalent number of PBs or *N_eq_* (see Materials and Methods) in the L33 and P33 forms were plotted for each residue (see Figure 5). These analyses confirmed that the three domains are very flexible because most residues can adopt more than one PB (*N_eq_*>1). Examining the absolute differences between L33 and P33 *N_eq_* (Δ*N_eq_*) showed significant alterations for several residues (Figure 5): I54 localized in the PSI domain; and Q438, A439, N452 in the I-EGF-1 domain; and Q485 in the I-EGF-2 domain. All of these residues were mainly localized on loops. These results revealed that the L33P substitution can significantly modify the structure of discrete areas within the three domains.

Regarding the PSI domain (Figure 5), the PB analyses revealed the presence of four small rigid areas (*N_eq_* = 1) interspersed in a highly flexible structure (*N_eq_*≥2). The L33 and P33 forms of the PSI had very similar *N_eq_* profiles. Surprisingly, residues L33 and P33 belong to a rigid stretch, encompassing residues 33, 34, and 35, which adopt only one PB, thus indicating a very rigid structure. Furthermore, PBs adopted by the residues 33 to 35 of the PSI domain are identical in the L33 and P33 forms (PBs *h*, *I* and *a*, Figure S4). Flanking residues from the C26–C38 loop (Figure 5 and Figure S4), which carry the L33/P33 residue, had a more flexible structure (*N_eq_*≥2).

PB analyses confirmed that the PSI, I-EGF-1, and I-EGF-2 domains have very flexible structures and that the L33P substitution can alter the structure of the PSI domain as well as the I-EGF-1 and I-EGF-2 domains. However, and rather surprisingly, the L33P substitution does not appear to modify the local structure of residues 33 to 35 in the C26–C38 loop.

## Discussion

### Dynamics of HPA-1a (L33) and HPA-1b (P33) β3 Structures

Using *in silico* 3D modeling of the L33 and P33 β3 forms, we compared the structural characteristics of the PSI, I-EGF-1, and I-EGF-2 domains. The static models were not sufficient to explain the clinical observations linked to the HPA-1 polymorphism. MD simulations were thus performed to better understand the structural modifications resulting from the L33P substitution. Although the αIIb subunit was not included in our analyses, β3 structures obtained in independent MD simulations were stable and did not significantly diverge from the initial β3 structure, as shown by RMSD values (Figure S2A). To be statistically informative, analyses were performed on five MD simulations representing a total of 2,749 structures for both β3 forms. The two β3 forms had high RMSF values (up to 6 Å) that revealed notable structural flexibility in the PSI, I-EGF-1, and I-EGF-2 domains, further confirmed by PCA. Interestingly, PCA also revealed that the P33 β3 form was more flexible, providing a first clue of structural differences.

Regarding the L33 and P33 relationships with the PSI, I-EGF-1, and I-EGF-2 domains, several differences were identified: (1) rASA analysis showed that high exposure (rASA >60%) is more frequent in L33; (2) inversely, P33 often makes a higher number of contacts with neighboring residues (>9), this difference being mainly due to fewer contacts between L33 and the I-EGF-2 domain; and (3) P33 can occupy positions close to the I-EGF-2 domain (center-of-mass distances <15 Å) apparently not accessible to L33.

The L33 residue is located in the middle of the C26–C38 loop of the PSI domain (Figure S5). The L33P substitution is expected to affect the loop structure, because a hydrophobic leucine is replaced by a hydrophilic proline [34] and because proline structure can induce sharp peptidic backbone reorientation [35]. Nonetheless and surprisingly, PB analyses demonstrated that residues 33 to 35 belong to a very rigid structure (*N_eq_* = 1) that is not affected by the L33P substitution. Local physical constraints involved in the L33P substitution are therefore compensated by structure modifications in the PSI as well as in the I-EGF-1 and I-EGF-2 domains, as shown by large *N_eq_* differences for some of their residues: i.e. I54 from the PSI domain, Q438, A439 and N452 from the I-EGF-1 domain and Q485 from the I-EGF-2 domain. None are localized at the interface with the αIIb subunit and are remote from the other domains. Their distances from L33/P33 revealed subtle allosteric alterations occurring throughout the PSI, I-EGF-1, and I-EGF-2 domains. These three domains compose the β3 knee and are involved in the β3 leg extension that occurs upon activation of the complex [12]. Structural modifications accompanying the L33P substitution may thus affect signal transduction. Interestingly, Q485 is localized in the disordered loop S474-Q485 where most of the movements of the β3 knee occur [12].

**Figure 6:**
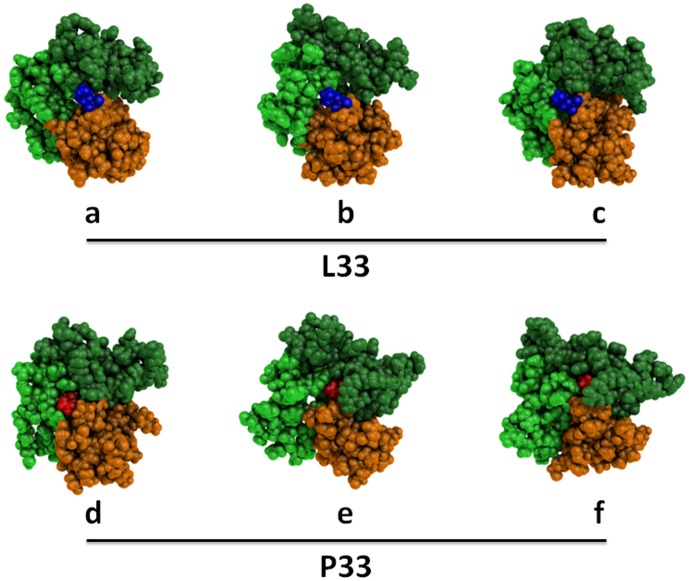
Snapshots of statistically representative structures of the PSI, I-EGF-1, and I-EGF-2 domains. Three structure snapshots of L33 (a, b, and c) and of P33 (e, f, and d) β3 were chosen among the statistically most frequent structures in terms of rASA and number of contacts. L33 and P33 atoms are shown as blue and red spheres, respectively. All remaining atoms belonging to the PSI, I-EGF-1, and I-EGF-2 domains are shown as orange, light green and dark green spheres, respectively. In these snapshots, L33 is largely exposed at the β3 surface while P33 can be remote. P33 is in relatively close contact with the I-EGF-2 domain, but L33 is not. These representations were generated using PyMOL software [16].

All these results show that the PSI, I-EGF-1, and I-EGF-2 domains have a very dynamic molecular structure, in which the L33P substitution displaces the equilibrium of their structure. Figure 6 illustrates the flexibility of the β3 knee of the L33 and P33 variants and their major structural differences. The PSI, I-EGF-1, and I-EGF-2 domains of three representative structures of L33 and P33 β3 forms were selected among the most frequent structures with regard to rASA and numbers of contacts (L33: rASA, 77% and number of contacts, 6; P33: rASA, 44% and number of contacts, 11). Striking differences were observed. L33 remained well exposed at the surface of β3, whereas P33 is frequently buried. P33 also showed higher mobility than L33 in the space defined by the three domains. However, these structures only reflect the most frequent, but transitory conformations.

### HPA-1a/-1b Structures and Immune Response

L33 and P33 β3 forms of the αIIbβ3 complex are both immunogenic and antigenic as they can result in fetal and neonatal alloimmune thrombocytopenia, but anti-HPA-1a alloimmunization is considered to be more frequent than that directed to HPA-1b [7]. Our results indicated that the L33P substitution did not significantly modify total ASA of the PSI domain, or the total ASA of the C26–C38 loop (≈770 Å^2^) (results not shown). Total ASA of both HPA-1 variants are compatible with the surface area of an antibody binding site (690 to 900 Å) [36]. Although still debated [37], several studies related to antigen 3D structures have suggested that surface exposure is a key characteristic of residues belonging to an epitope [38], [39]. Comparing residues from a set of known antigen 3D structures, Liu and Hu [39] reported that in most epitopes, leucine and proline have rASA values greater than 40% and 50%, respectively. In the same study, lower flexibility, evaluated using the B-factor, seems to characterize residues involved in epitopes. These observations correlate with our MD simulation results, which generally showed higher rASA values for L33 (≈70%) than for P33 (≈44%), and increased flexibility of the PSI, I-EGF-1, and I-EGF-2 domains in the P33 variant. Such structural differences may modify β3 antigenicity and possibly induce differences in the immune response to the HPA-1a and HPA-1b alleles. As far as we know, the high specific HPA-1a immunogenicity is attributed to the ability of the processed L33 β3 peptides to bind, with high affinity, the HLA-DRB3*0101 allele carried by the antigen-presenting cells [40]–[42]. However, although our study shows clear structural differences between the two β3 variants that may affect the antigenicity, we cannot conclude as to immunogenicity.

Barron-Casella et al. [43] showed that the 50 N-terminal β3 residues carry the anti-HPA-1a epitopes with key amino acids 30, 32, 33 and 39. Accordingly, our MD simulations showed that residues 30, 32 and 33 are highly exposed (rASA>60%) on the surface of the PSI domain (data not shown). However, the mean rASA of residue 39 was 27±11%, indicating significantly lower surface exposure. This residue may be necessary for correct epitope presentation without being directly involved [37]. The L33P substitution did not affect the rASA of residues 30, 32, and 39 (not shown).

Several studies [5], [44], [45] have reported that anti-HPA-1a alloantibodies are directed to different epitopes and can be divided into two groups. In the type I group, binding of anti-HPA-1a alloantibodies is not affected by β3 alterations such as C13–C435 disulfide reduction [5], partial deletions [44], and the L33V substitution [45] while in the type II group, intact β3 structure is required. Our MD simulation results support the hypothesis of multiple epitopes, because total ASA (4,000 Å^2^) for the PSI domain is compatible with multiple antibody binding sites (690 to 900 Å^2^) [36]. Moreover, residues from the adjacent domains I-EGF-1 and I-EGF-2 can also participate in HPA-1a epitopes. The L33P substitution was shown to modify not only the PSI domain structure, but also the structures of I-EGF-1 and I-EGF-2, potentially increasing the availability of various discontinuous epitopes.

### HPA-1a/-1b Structural Insights and Thrombotic Risk

Experiments designed to establish a link between the HPA-1b β3 variant and a thrombosis risk have yielded contradictory results [10], [46]–[49]. Nonetheless, some studies suggest that the L33P substitution neither increases complex affinity nor maximum binding for soluble fibrinogen [48], [50] but may facilitate adhesion to fibrinogen [48], [49]. Vijayan and Bray [10] suggest that β3 structural modifications linked to the L33P substitution favor leg separation or provide more stability to the open-head conformation after ligand binding. Our study shows that the substitution modifies the local structural equilibrium of the PSI, I-EGF-1, and I-EGF-2 domains and, interestingly, a proline in position 33 increases the structural flexibility of the three domains that participate in the knee of the β3 stalk. Adhesion to surface-bound fibrinogen does not require activation [51] and the mechanism of binding by the unactivated form of αIIbβ3 remains to be determined. Nonetheless, a recent study performed on αLβ2 suggests that integrin binding to adsorbed ligands also requires αβ legs extension and separation through movements at the interface between the I-EGF-1 and PSI domains and the I-EGF-2 domain [52]. Consequently, the increase in structural flexibility of the P33 PSI, I-EGF-1, and I-EGF-2 domains would affect these mechanisms. How to link a gain of flexibility with an increase of platelet adhesiveness? Our study does not intend to fill in this gap, nonetheless it can be hypothesized that the gain of flexibility would destabilize the “bent” form of αIIbβ3 and/or facilitate legs extension, “preparing” the P33 form of the complex to adhere onto substrate.

### Conclusion

To compare structural effects of the HPA-1 variants, we built a complete 3D model of the β3 subunit extracellular domain. *In silico* mutageneses were performed to obtain 3D models of L33 and P33 β3 forms, and MD simulations were run to highlight key structural differences. Analyses of MD simulations showed that the PSI, I-EGF-1, and I-EGF-2 domains are highly flexible and mobile. Unexpectedly, the L33P substitution did not alter the very local structure (residues 33 to 35) of the C26–C38 loop of the PSI domain, but instead resulted in modifications of the structural equilibrium of the PSI, I-EGF-1, and I-EGF-2 domains. These MD simulations therefore provide a novel structural explanation of epitope complexity in the HPA-1 alloimmune system. Counter-intuitively, proline at position 33 introduces higher structure flexibility than leucine in the β3 knee defined by the PSI, I-EGF-1, and I-EGF-2 domains. This specific increase in flexibility may explain the increased adhesion capacity of HPA-1b platelets to fibrinogen and the possible thrombotic risk associated with the HPA-1b phenotype. Our study thus provides new key insights into the relationship between HPA-1 polymorphism and β3 structure and its possible effects on alloimmune response and platelet function.

## Supporting Information

Figure S1 **Electrostatic surface of the L33 (HPA-1a) and P33 (HPA-1b) allelic forms of β3.** Computed electrostatic maps projected on the van der Waals molecular surfaces of the models are viewed from the β3 knee side of the glycoprotein. Zoomed images of the polymorphic area are also shown. Residues L33 and P33 are shown (green dot). Negative, neutral, and positive electrostatic charges are shown in red, white and blue, respectively. The L33P substitution does not induce any significant changes in electrostatic charge. These representations were generated using PyMOL software (DeLano WL (2002) The PyMOL Molecular Graphics System, Version 1.5.0.4 Schrödinger, LLC.).
(TIF)

Figure S2 **RMSD of the PSI, I-EGF-1 and I-EGF-2 domains.** (A) Calculated root mean square deviations (RMSD) are individually presented for the four MD simulations of 50 ns and the fifth of 100 ns for the L33 and P33 forms of the β3 subunit (blue and red lines, respectively). The first 5 ns of each simulation, i.e. the time to reach stability, were omitted. RMSD values were stable throughout the MD simulations. (B) RMSD frequencies calculated from all MD simulations for the L33 and P33 β3 forms are shown (blue and red lines, respectively). P33 RMSD (mean 7.2±1.0 Å) is higher than L33 RMSD (mean 4.7±0.7 Å), suggesting a greater shift from the starting structures.
(TIF)

Figure S3 **Frequency of Cα number of contacts with each domain.** Frequency of Cα number of contacts of L33 and P33 (panels A and B, respectively) with atoms from the PSI, I-EGF-1 and I-EGF-2 domains are shown in orange, black and purple, respectively. The sum of all contacts for L33 (blue line) and P33 (red line) are shown. While number of contacts did not vary for the PSI domain of the two β3 forms, the I-EGF-2 (purple line) domain shows that P33 frequently makes a high number of contacts (≥4) with the I-EGF-2 domain that are not observed for L33. However, the number of contacts with the I-EGF-1 domain did not vary significantly between the L33 or P33 β3 forms.
(TIF)

Figure S4 **Protein Block analyze of the C26–C38 loop.** These panels show the PBs adopted by each residue of the C26–C38 loop in the L33 and P33 forms of β3 subunit. A color scale from dark blue (0%) to red (100%) indicates the proportion of each PB adopted by a residue. Leucine or proline at position 33 adopted a single structure for its backbone structure (PB *h*). Single PBs are also observed for residues 34 and 35 (respectively *i* and *a*) whatever the amino acid (leucine or proline) in position 33. Structures adopted by the remaining residues are also very similar although they differ slightly in terms of frequencies. The C26–C38 loop structure is not affected by the L33P substitution.
(TIF)

Figure S5 **Structure of the C26–C38 loop.** The L33P polymorphism is located in the middle of the C26–C38 loop. Note the presence of a proline in position 32. This representation was generated using PyMOL software (DeLano WL (2002) The PyMOL Molecular Graphics System, Version 1.5.0.4 Schrödinger, LLC.).
(TIF)
