# Correction: Modeling and Molecular Dynamics of HPA-1a and -1b Polymorphisms: Effects on the Structure of the β3 Subunit of the αIIbβ3 Integrin

**DOI:** 10.1371/annotation/55a1f8de-c479-4227-90c0-f2fa0d07ae2b

**Published:** 2013-02-12

**Authors:** Vincent Jallu, Pierre Poulain, Patrick F. J. Fuchs, Cecile Kaplan, Alexandre G. de Brevern

The following information was missing from the Funding section: This work was granted access to HPC resources at the French National Computing Center CINES under grant no. 2011-c2011076723 funded by the GENCI (Grand Equipement National de Calcul Intensif).

