# Supplementary figures and images for: Modeling and Molecular Dynamics of HPA-1a and -1b Polymorphisms: Effects on the Structure of the β3 Subunit of the αIIbβ3 Integrin
Source: PLoS One. 2012 Nov 14;7(11):e47304. doi: 10.1371/journal.pone.0047304 (PMC3498292; doi:10.1371/journal.pone.0047304)

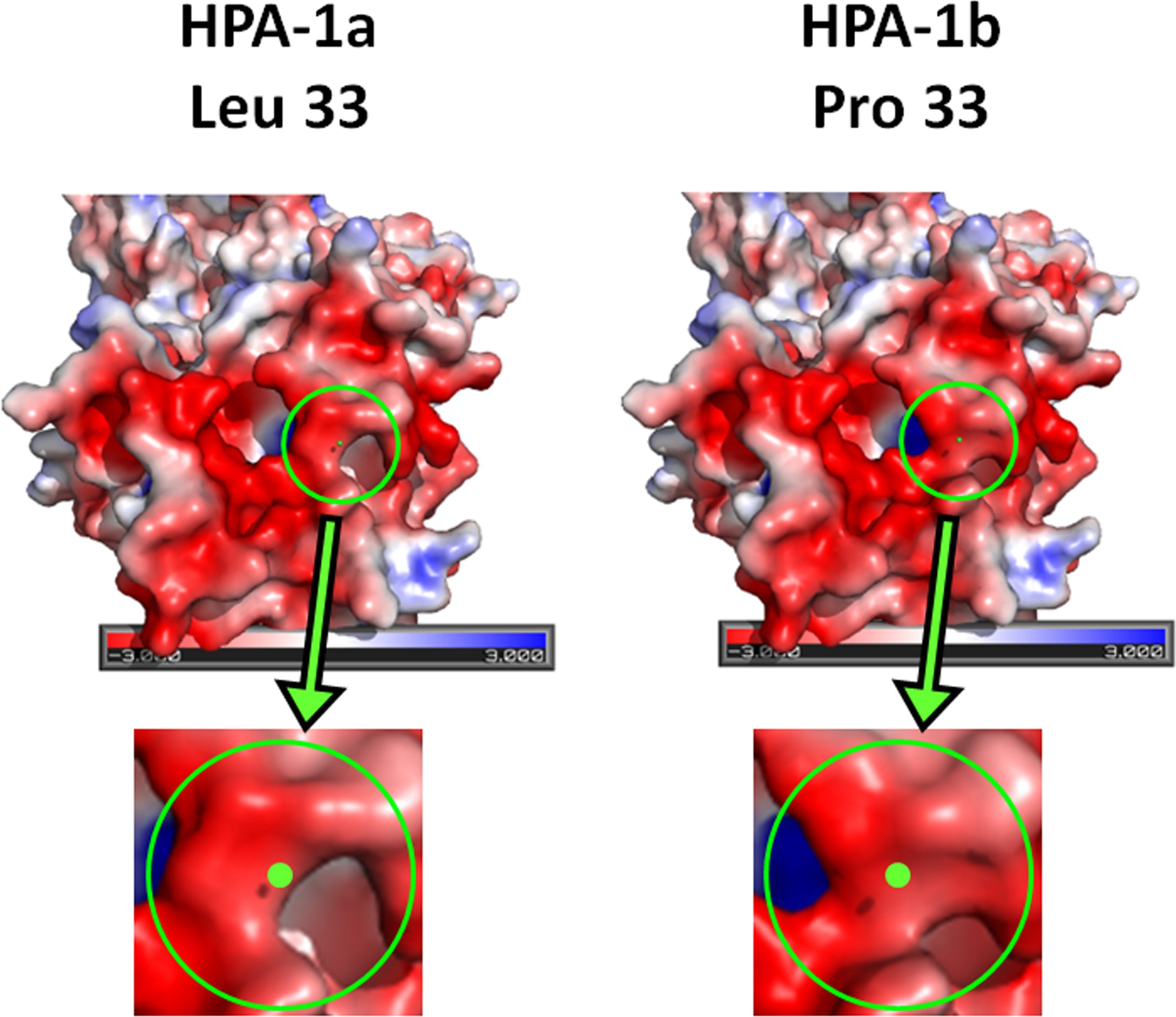

Supplement: Figure S1 — Electrostatic surface of the L33 (HPA-1a) and P33 (HPA-1b) allelic forms of β3. Computed electrostatic maps projected on the van der Waals molecular surfaces of the models are viewed from the β3 knee side of the glycoprotein. Zoomed images of the polymorphic area are also shown. Residues L33 and P33 are shown (green dot). Negative, neutral, and positive electrostatic charges are shown in red, white and blue, respectively. The L33P substitution does not induce any significant changes in electrostatic charge. These representations were generated using PyMOL software (DeLano WL (2002) The PyMOL Molecular Graphics System, Version 1.5.0.4 Schrödinger, LLC.). (TIF) [file pone.0047304.s001.tif]

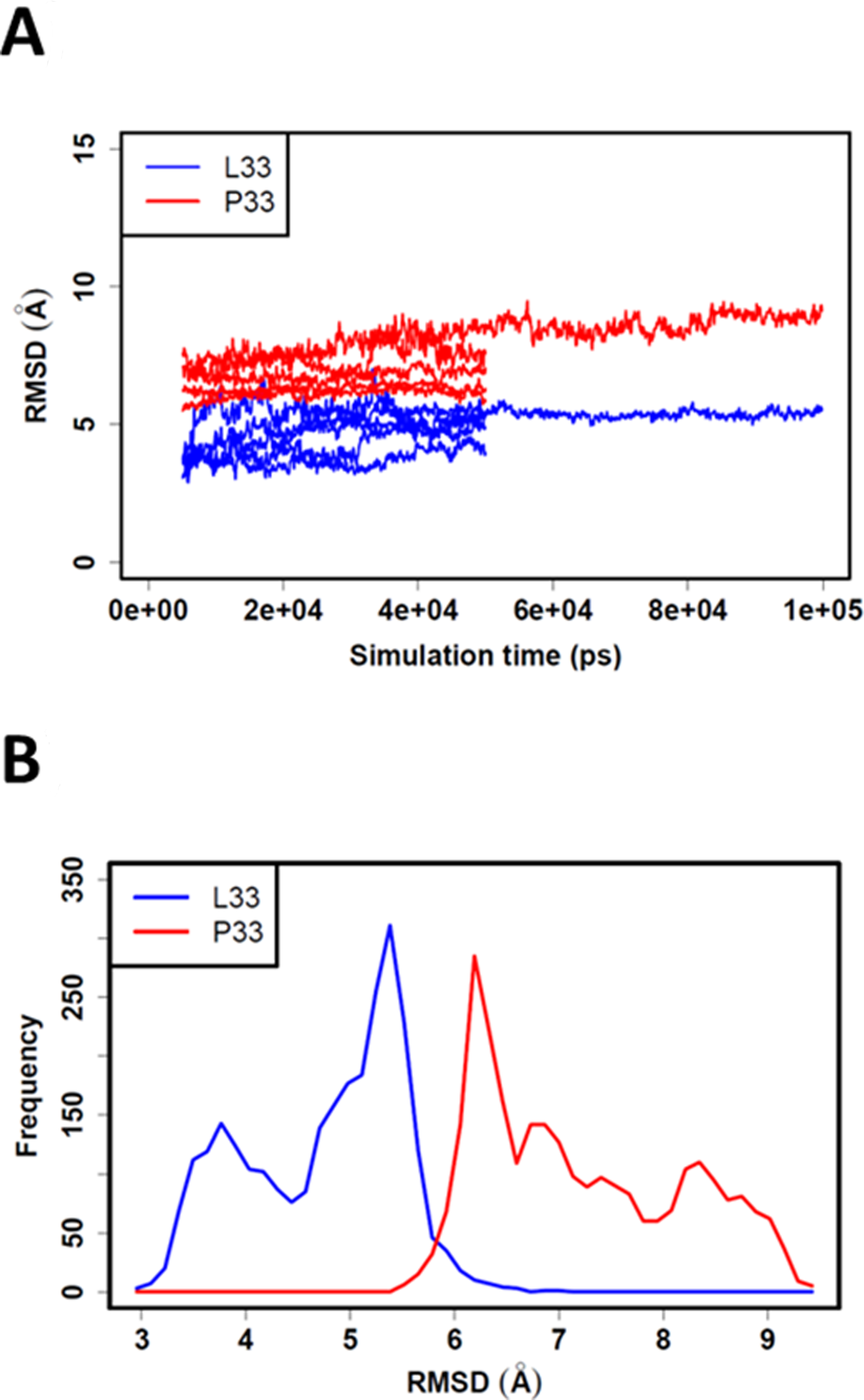

Supplement: Figure S2 — RMSD of the PSI, I-EGF-1 and I-EGF-2 domains. (A) Calculated root mean square deviations (RMSD) are individually presented for the four MD simulations of 50 ns and the fifth of 100 ns for the L33 and P33 forms of the β3 subunit (blue and red lines, respectively). The first 5 ns of each simulation, i.e. the time to reach stability, were omitted. RMSD values were stable throughout the MD simulations. (B) RMSD frequencies calculated from all MD simulations for the L33 and P33 β3 forms are shown (blue and red lines, respectively). P33 RMSD (mean 7.2±1.0 Å) is higher than L33 RMSD (mean 4.7±0.7 Å), suggesting a greater shift from the starting structures. (TIF) [file pone.0047304.s002.tif]

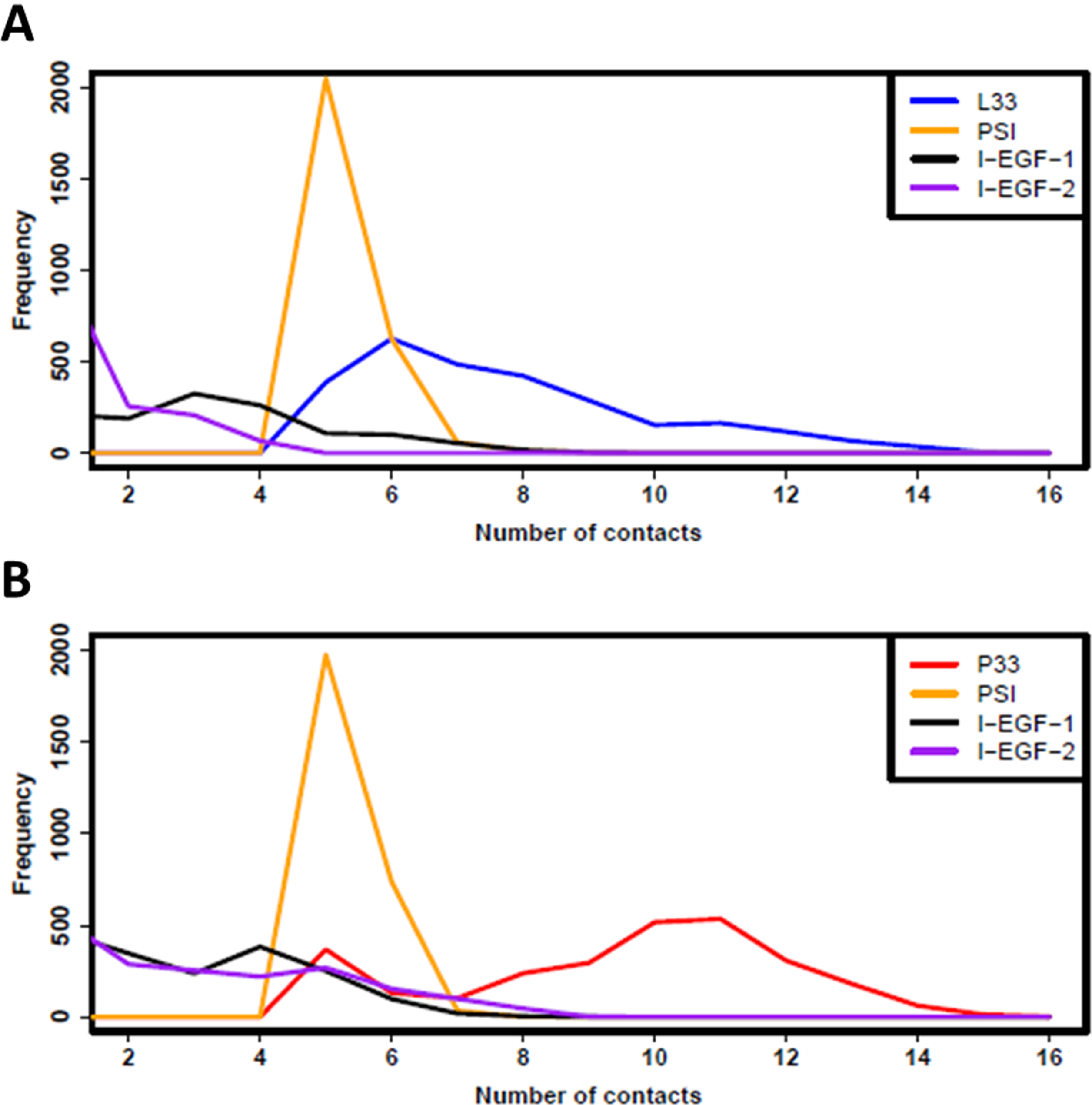

Supplement: Figure S3 — Frequency of Cα number of contacts with each domain. Frequency of Cα number of contacts of L33 and P33 (panels A and B, respectively) with atoms from the PSI, I-EGF-1 and I-EGF-2 domains are shown in orange, black and purple, respectively. The sum of all contacts for L33 (blue line) and P33 (red line) are shown. While number of contacts did not vary for the PSI domain of the two β3 forms, the I-EGF-2 (purple line) domain shows that P33 frequently makes a high number of contacts (≥4) with the I-EGF-2 domain that are not observed for L33. However, the number of contacts with the I-EGF-1 domain did not vary significantly between the L33 or P33 β3 forms. (TIF) [file pone.0047304.s003.tif]

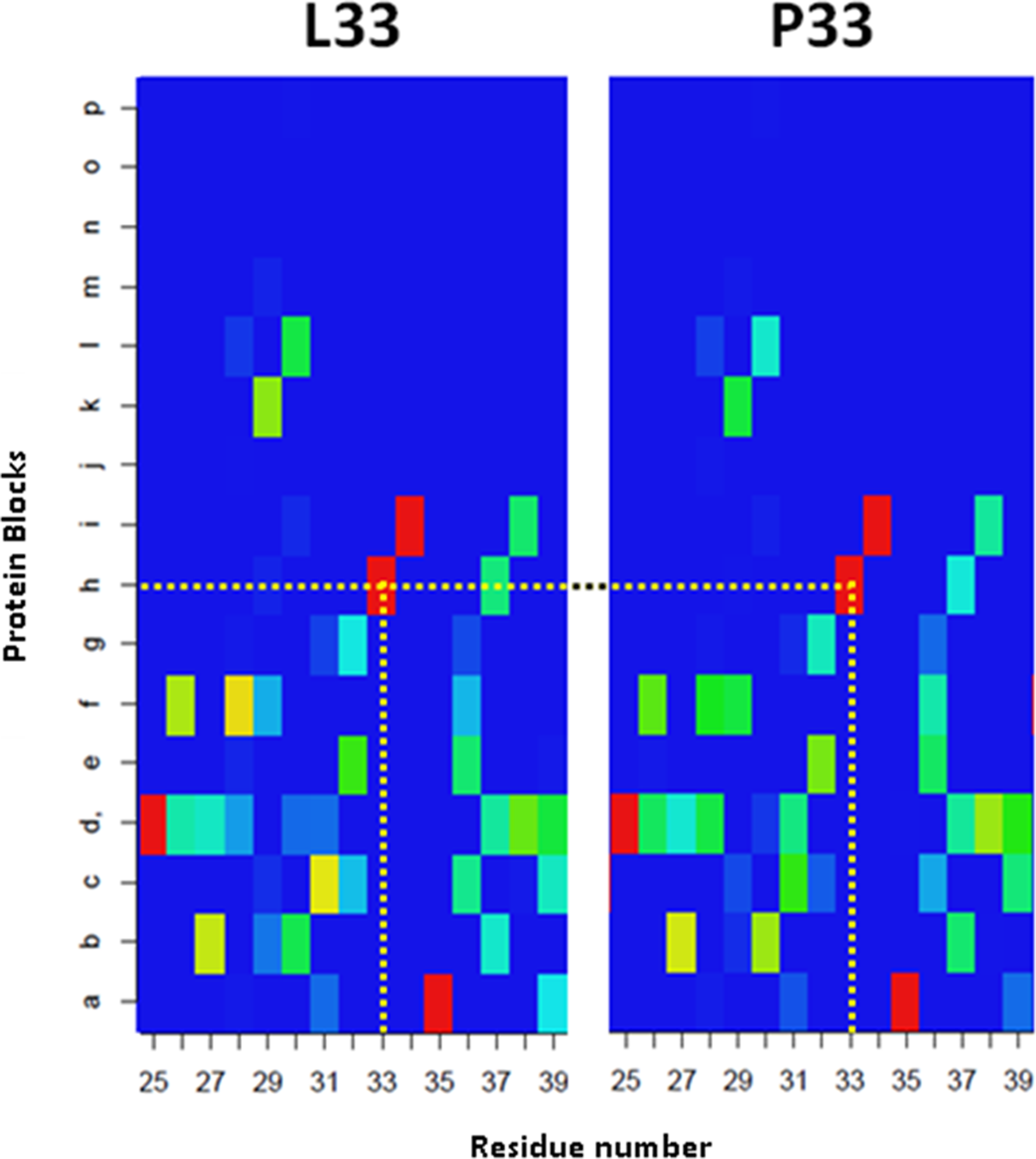

Supplement: Figure S4 — Protein Block analyze of the C26–C38 loop. These panels show the PBs adopted by each residue of the C26–C38 loop in the L33 and P33 forms of β3 subunit. A color scale from dark blue (0%) to red (100%) indicates the proportion of each PB adopted by a residue. Leucine or proline at position 33 adopted a single structure for its backbone structure (PB h). Single PBs are also observed for residues 34 and 35 (respectively i and a) whatever the amino acid (leucine or proline) in position 33. Structures adopted by the remaining residues are also very similar although they differ slightly in terms of frequencies. The C26–C38 loop structure is not affected by the L33P substitution. (TIF) [file pone.0047304.s004.tif]

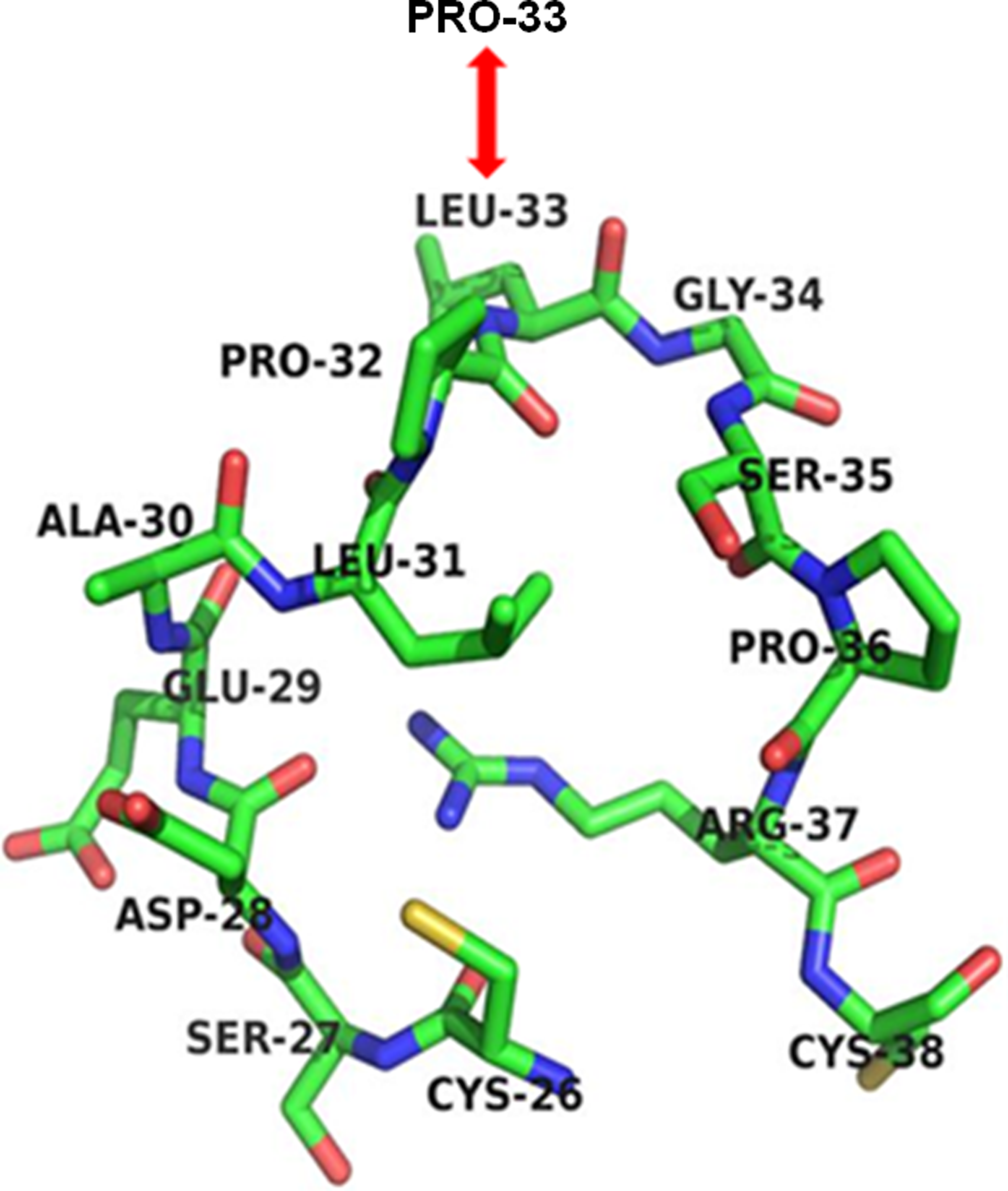

Supplement: Figure S5 — Structure of the C26–C38 loop. The L33P polymorphism is located in the middle of the C26–C38 loop. Note the presence of a proline in position 32. This representation was generated using PyMOL software (DeLano WL (2002) The PyMOL Molecular Graphics System, Version 1.5.0.4 Schrödinger, LLC.). (TIF) [file pone.0047304.s005.tif]
